# Pseudocirrhosis in Breast Cancer – Experience From an Academic Cancer Center

**DOI:** 10.3389/fonc.2021.679163

**Published:** 2021-07-02

**Authors:** Dharmesh Gopalakrishnan, Ain Shajihan, Andrei S. Purysko, Jame Abraham

**Affiliations:** ^1^ Department of Medical Oncology, Roswell Park Comprehensive Cancer Center, Buffalo, NY, United States; ^2^ College of Medicine, Northeast Ohio Medical University, Rootstown, OH, United States; ^3^ Section of Abdominal Imaging, Imaging Institute, Cleveland Clinic, Cleveland, OH, United States; ^4^ Department of Hematology and Medical Oncology, Taussig Cancer Institute, Cleveland Clinic, Cleveland, OH, United States

**Keywords:** pseudocirrhosis, breast cancer, portal hypertension, hepatocellular failure, liver metastases

## Abstract

**Background:**

Pseudocirrhosis is characterized by radiological changes in the liver that resemble cirrhosis, but with more rapid onset and progression. Though reported most frequently in patients with metastatic breast cancer, little is known about its prognostic factors and impact on breast cancer outcomes.

**Methods:**

In this observational study, we reviewed abdominal CT and/or MRI scan reports of all patients with invasive breast cancer diagnosed at our center, during a ten-year period, to identify patients with pseudocirrhosis. Exclusion criteria included lack of baseline imaging, pre-existing cirrhosis, hepatitis B or C, other chronic liver diseases, or heavy alcohol use. Routine descriptive statistical measures were used. Survival distributions were estimated using Kaplan-Meier method, and Cox regression was used for multivariate analysis. Two-tailed *p* < 0.05 was considered significant.

**Results:**

Eighty-six patients were included – all were females, median age was 57.5 years, and 90% were Caucasian; 86% of primary tumors were hormone-receptor positive and 17% were HER2 positive. Most patients (98%) had metastatic disease with liver involvement (94%), and were heavily pre-treated – 97% with chemotherapy, 85% with hormonal therapy, and 19% with anti-HER2 agents. Median interval from breast cancer diagnosis to pseudocirrhosis was 75.4 months (IQR 35.2-115.3 months). Thirty-six percentage of patients had ≥1 signs of portal hypertension and 49% had ≥1 signs of hepatocellular failure. Pseudocirrhosis led to permanent discontinuation of chemotherapy, endocrine therapy, and all systemic therapies in 29%, 31%, and 20% patients, respectively. Median overall survival from diagnosis of pseudocirrhosis was 10.0 months (95%CI 5.2-14.8 months). On multivariate analysis, coagulopathy, hyperbilirubinemia, hypoalbuminemia, and cancer progression were independently predictive of mortality.

**Conclusions:**

In this largest series, to date, of breast cancer with pseudocirrhosis, the latter was often complicated by portal hypertension and hepatocellular failure, and markedly impacted breast cancer management. Survival was shorter for patients who developed hepatocellular failure.

## Highlights

- We report the largest series, to date, of patients with pseudocirrhosis complicating breast cancer.
- Pseudocirrhosis occured predominantly in patients with liver metastases exposed to multiple lines of systemic therapy, and was not restricted to any particular biological subtype of breast cancer.
- It was frequently complicated by signs of portal hypertension and hepatocellular failure, and often adversely impacted the ability of patients to tolerate systemic therapies for breast cancer.
- Clinically overt hepatocellular failure was predictive of increased mortality while portal hypertension was not.

## Introduction

Hepatic pseudocirrhosis, previously known as *hepar lobatum*, is characterized by radiological features that are often indistinguishable from true cirrhosis (*i.e.* irregular contour with nodularity, caudate lobe hypertrophy, capsular retraction, and/or segmental/lobar volume loss), and can be complicated by portal hypertension and hepatocellular failure (1–4). However, this under-recognized and under-reported entity is typified by a distinctly more rapid clinical course as well as the absence of bridging fibrosis between regenerating nodules on histological examination (1, 5). Though pseudocirrhosis was initially described almost exclusively in patients with breast cancer and liver metastases in the setting of systemic therapies, isolated cases have subsequently been reported in non-metastatic breast as well as colorectal, esophageal, pancreatic, ovarian, and thyroid cancers (6–10).

Multiple series have reported on the clinical characteristics of pseudocirrhosis, though sample sizes have been relatively small. Also, there is paucity of data regarding its complications and prognosis as well as implications for systemic antineoplastic therapies. We conducted a single-institution analysis comprising patients who developed pseudocirrhosis in the setting of breast cancer, aimed at describing its clinical features, complications, prognostic determinants, and impact on systemic breast cancer therapies.

## Methods

### Patients

**Figure 1:**
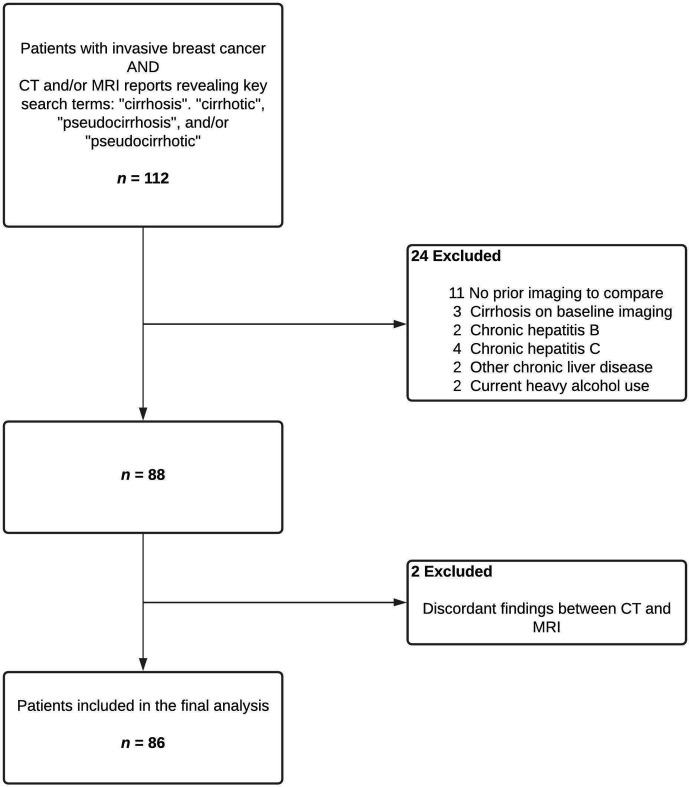
Flow diagram depicting selection of the study population.

In this retrospective, observational study, we reviewed abdominal computed tomography (CT) and/or magnetic resonance imaging (MRI) reports of all patients with invasive breast cancer diagnosed during at 10-year period from 2007 to 2016. Patients who were reported to have cirrhotic liver morphology on CT and/or MRI were first identified using key search terms as listed in **Figure 1**. Relevant images were reviewed by the study team to confirm the findings. We then excluded patients who either had cirrhosis on pre-treatment imaging or lacked baseline CT or MRI of the liver for comparison. Patients with history of heavy alcohol use, hepatitis B, hepatitis C, or pre-existing chronic liver disease due to other etiologies were also excluded. Cases where CT and MRI findings were discordant for evidence of pseudocirrhosis were excluded from analyses, though these discrepancies could have been related to interobserver variability or ascites-related artifacts in MRI of the liver. Patients were not included if they only had carcinoma *in situ* of the breast with no evidence of invasive cancer.

### Statistical Analysis

Descriptive measures consisted of frequencies and percentages for all categorical variables. Continuous variables were summarized using median with interquartile range (IQR) for non-parametric data. Hyperbilirubinemia was defined as total bilirubin level greater than the upper limit of normal (ULN) reference range. Similarly, hypoalbuminemia was defined as serum albumin level below the lower limit of normal (LLN). Coagulopathy was defined as new prolongation in the prothrombin time and/or partial thromboplastin time above the ULN. For the purposes of this study, portal hypertension was defined as the detection of one or more of the following: new splenomegaly (>12 cm in cranial caudal length), ascites with high (≥ 1.1 g/dL) serum-ascites albumin gradient (SAAG), and varices on upper endoscopy and/or cross-sectional imaging. Hepatocellular failure was defined as the presence of one or more of the following: direct hyperbilirubinemia, new coagulopathy, and hepatic encephalopathy.

Survival distributions were estimated using Kaplan-Meier method, and patients who were alive at last follow-up were censored. Cox proportional hazards regression with forward model selection was used to investigate the association between multiple predictor variables and survival outcomes. Hazard ratios (HR) with 95% confidence intervals (CI) were reported for significant covariates. The survival endpoints of interest in this study were overall survival (OS) and breast cancer-specific survival (BCSS). OS was defined as time from the first identification of pseudocirrhosis to death from any cause, and BCSS as time to death from causes directly related to breast cancer. A nominal two-tailed significance level of *p* < 0.05 was chosen for statistical significance. SPSS version 22 was used to perform all statistical analyses.

### Compliance With Ethical Standards

The study protocol was approved by our Institutional Review Board (IRB) and requirement for informed consent was waived after it was determined the study involves no more than minimal risk to the subjects.

## Results

### Demographics

We identified 86 patients satisfying the inclusion and exclusion criteria (**Figure 1**). All the study subjects were females and their median age was 57.5 years (IQR 51.0 – 68.6 years, range 32.4 – 82.4 years). Seventy-seven (90%) patients were Caucasian and 7 (8%) were African American (**Table 1**).

**Table 1:** Baseline characteristics of breast cancer (n = 86).

Feature	*n* (%)*
**Receptor status**	
Estrogen-receptor positive	72 (83.7)
Progesterone-receptor positive	50 (58.1)
HER2 positive	15 (17.4)
**Histology**	
Invasive ductal carcinoma (IDC)	58 (67.4)
Invasive lobular carcinoma (ILC)	16 (18.6)
IDC + ILC	6 (7.0)
Grade 1	5 (5.8)
Grade 2	34 (39.5)
Grade 3	29 (33.7)
**Disease extent**	
Stage IV	84 (97.7)
Liver metastases +	81 (94.2)
1-3 liver metastases	1 (1.2)
4-10 liver metastases	5 (5.8)
≥ 11 liver metastases	75 (87.2)
**Prior systemic therapies**	
**Endocrine therapy**	73 (84.9)
Tamoxifen	44 (51.2)
Median duration (months)	35 (16 - 60)
Aromatase inhibitor	60 (69.8)
Median duration (months)	35 (16 - 57)
CDK 4/6 inhibitor	47 (54.6)
**Anti-HER2 therapy**	16 (18.6)
Median duration (months)	15 (6 - 22)
Trastuzumab	16 (18.6)
Pertuzumab	11 (12.8)
TDM1	6 (7.0)
**Chemotherapy**	83 (96.5)
Median number of lines	3 (range 1-10)
Anthracycline	50 (58.1)
Taxane	75 (87.2)
Platinum	6 (7.0)
Cyclophosphamide	52 (60.5)

*Unless specified in parentheses.

### Breast Cancer – Disease Characteristics and Treatments

Overall, 74 (86%) primary tumors were hormone-receptor positive (HR^+^) and 15 (17%) were HER2 positive (HER2^+^) – 65 (76%) were HR^+^/HER2^-,^ 9 (10%) were HR^+^/HER2^+^, 6 (7%) were HR^-^/HER2^+^, and 6 (7%) were HR^-^/HER2^-^. Fifty-eight (67%) were invasive ductal carcinoma and 22 (26%) had invasive lobular or mixed histology; 62 (73%) tumors were grade 2 or 3 (**Table 1**). At initial diagnosis of breast cancer, 45 (53%) patients had stage I or II, 17 (20%) had stage III, and 21 (24%) had stage IV disease. The median interval from breast cancer diagnosis to the detection of pseudocirrhosis was 75.4 months (IQR 35.2-115.3 months), while the median interval from metastases to pseudocirrhosis was 23.4 months (IQR 9.3-47.0 months). Eighty-four (98%) patients had stage IV disease at the time of diagnosis of pseudocirrhosis with a median of three (range 1 -6) metastatic sites. Eighty-one (94%) patients had liver metastases, 75 (88%) had >10 liver lesions, and the median size of largest reported liver metastasis was 35 mm (IQR 22.5 – 57 mm). The median duration from initial detection of liver metastases to pseudocirrhosis was 13.3 months (IQR 5.8-25.8 months).

Seventy-three (85%) patients were treated with endocrine therapy for a median duration of 35 months (IQR 16.25 – 60), and 16 (19%) received anti-HER2 therapy for a median duration of 15 months (IQR 6.5 – 22.5). Eighty-three (97%) patients were treated with chemotherapy – 50 (58%) with anthracyclines, 75 (87%) with taxanes, and 6 (7%) received a platinum agent (**Table 1**).

### Pseudocirrhosis – Manifestations and Outcomes

**Figure 2:**
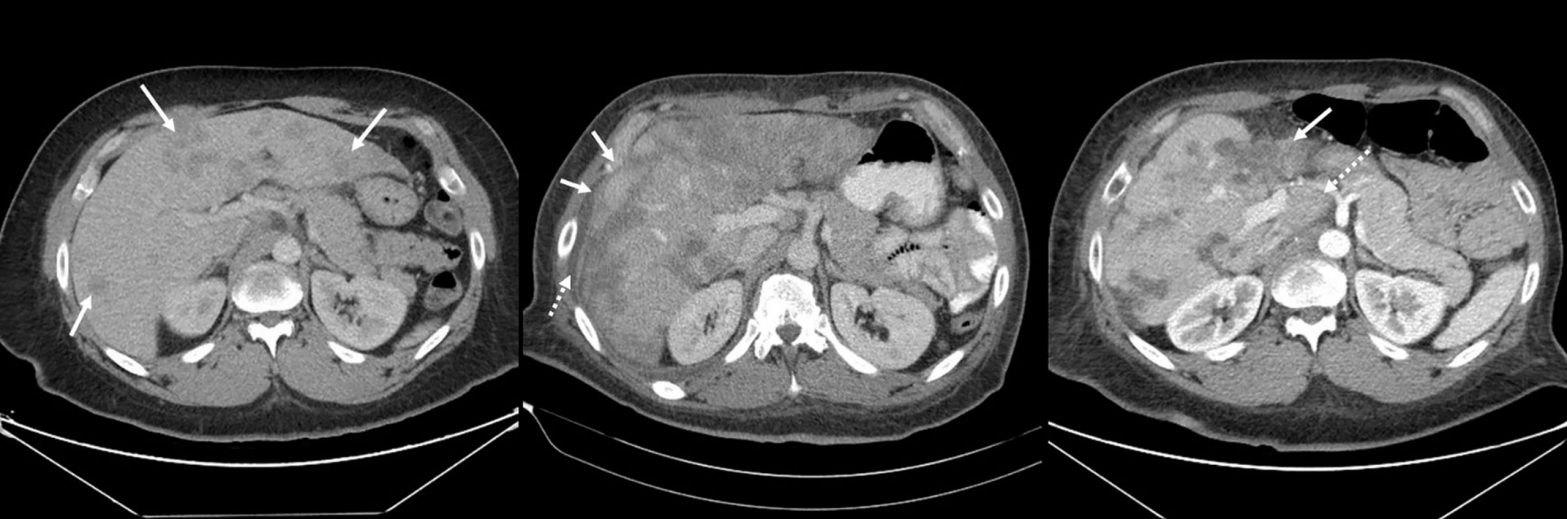
Serial contrast enhanced CT images of the abdomen in the axial plane showing evolution of pseudocirrhosis in a patient with breast cancer and liver metastases. **(A)** Baseline image shows multiple liver metastases (arrows); **(B)** At 6 months follow up, the lesions became larger and more confluent and the hepatic contour started to become nodular (solid arrows). A small volume of perihepatic ascites started to accumulate (dashed arrow). **(C)** At 13 months, there was significant atrophy of the left lobe (solid arrow) and hypertrophy of the caudate lobe (dashed arrow).

The most frequent findings on liver imaging were nodular contour (98%) and capsular retraction (83%) (**Figure 2**). Median duration of follow-up from the diagnosis of pseudocirrhosis was 28.1 months (95% CI 10.9 – 45.2 months). During this period, 31 (36%) patients developed one or more signs of portal hypertension – new splenomegaly in 13 (15%), esophageal and/or gastric varices in 16, upper gastrointestinal bleeding from esophageal varices in 8 (9%), and ascites with high SAAG in 30 (35%). Forty-two (49%) patients developed one or more signs of hepatocellular failure during follow-up – direct hyperbilirubinemia in 41 (48%), hepatic encephalopathy in 12 (14%), and new-onset of coagulopathy in 26 (30%) (**Table 2**).

**Table 2:** Complications and other characteristics of pseudocirrhosis (n = 86, unless specified).

Feature	*n* (%)
**Radiological findings**	
Nodular hepatic contour	84 (97.7)
Capsular retraction	71 (82.6)
Caudate lobe enlargement	58 (67.4)
Segmental or lobar volume loss	53 (61.6)
**Signs of portal hypertension**	31 (36.0)
New splenomegaly (>12 cm)	13 (15.1)
Esophageal and/or gastric varices	16 (NAφ)
Variceal gastrointestinal bleeding	8 (9.3)
High albumin-gradient ascites	30 (34.9)
**Signs of hepatocellular failure**	42 (48.8)
Direct hyperbilirubinemia	41 (47.7)
Hepatic encephalopathy	12 (14.0)
New coagulopathy	26 (30.2)
Breast cancer response*	
Complete response (CR)	2 (2.3)
Partial response (PR)	13 (15.1)
Stable disease (SD)	24 (27.9)
Progressive disease (PD)	46 (53.5)
Not assessed	1 (1.2)
Status of liver metastases*	
Enlarging and/or increasing	33 (38.4)
Shrinking and/or decreasing	25 (29.1)
Stable or mixed response	19 (22.1)
**Change in breast cancer therapies^¶^**	
Discontinued chemotherapy	21/73 (28.8)
Discontinued endocrine therapy	13/42 (30.9)
Transitioned to hospice	17/86 (19.8)

φ denominator unknown; * at the time of diagnosis of pseudocirrhosis; ^¶^ within 4 weeks of diagnosis of pseudocirrhosis.

At the time of first report of pseudocirrhosis, 46 (54%) patients had disease progression, 24 (28%) had stable disease, 13 (15%) had partial response, and only 2 (2%) patients were in a complete response (2%). Responses within liver metastases were also heterogeneous – 33 (38%) patients had enlarging liver lesions, 19 (22%) had stable, and 25 (29%) had shrinking lesions when pseudocirrhosis was first reported. Forty-two (49%) patients required a change in systemic therapy within 4 weeks of diagnosis of pseudocirrhosis - 40 (47%) required a change in chemotherapy and 16 (19%) required a change in endocrine therapy. Reasons for change in systemic therapy included hepatocellular failure in 26 (62%) patients and progression of breast cancer in 33 (79%) patients. Seventeen (20%) patients had to be transitioned to only best supportive care (**Table 2**).

**Figure 3:**
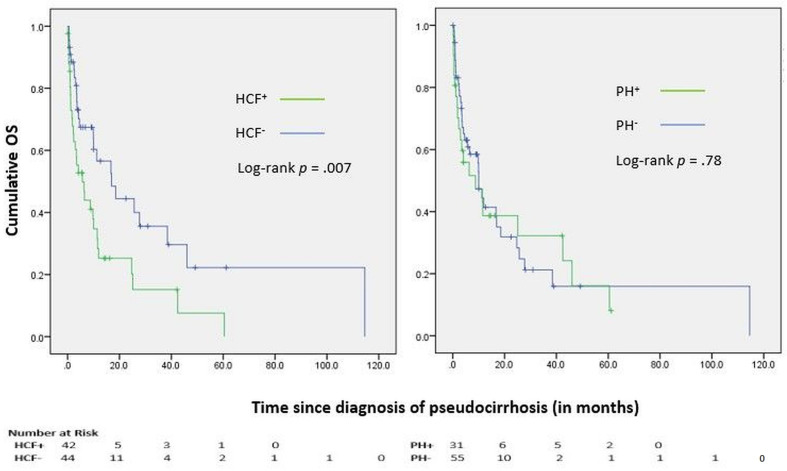
Kaplan-Meier curves for Overall Survival (OS); HCF, Hepatocellular failure; PH, Portal hypertension.

Median OS (mOS) from the diagnosis of pseudocirrhosis was 10.0 months (95%CI 5.2-14.8 months), with a 12-month OS of 41%. OS was shorter for patients who developed hepatocellular failure compared to those who had preserved liver function (mOS of 5.7 *vs* 16.9 months, HR 0.48 (0.28-0.83), log-rank p = .007) (**Figure 3**). However, OS was comparable among patients with and without signs of portal hypertension (mOS of 8.8 *vs* 10.0 months, HR 1.08 (0.62-1.89), log-rank p = .78) (**Figure 3**). A multivariate Cox proportional hazards model revealed that new-onset coagulopathy (HR of 3.4, 95% CI 1.3-9.1, log-rank p = .01), hyperbilirubinemia (HR 2.8, 95% CI 1.2-6.3, log-rank p = .01), hypoalbuminemia (HR 2.8, 95% CI 1.02-7.58, log-rank p = .047), and breast cancer progression (HR 10.5, 95% CI 3.5-31.9, log-rank p <.001) were significant predictors of all-cause mortality. Notably, age, race, hormone receptor or HER2 status, and the number, size or status of liver metastases were not predictive of survival. Median OS from the diagnosis of breast cancer and distant metastases were 108.4 months (IQR 89.8-127.1 months) and 50.8 months (IQR 34.0-67.6 months), respectively.

Median BCSS was 11.6 months (95% CI 4.7 – 18.4 months). On multivariate analysis, coagulopathy (HR of 3.2, 95% CI 1.1 – 9.2, log-rank p = .03), hyperbilirubinemia (HR 3.2, 95% CI 1.3 – 7.9, log-rank p = .01), and breast cancer progression (HR 12.1, 95% CI 3.5 – 41.5, log-rank p <.001) were significant predictors of breast cancer-related mortality.

## Discussion

To our knowledge, this is the largest reported series of pseudocirrhosis in breast cancer. Patients in this study were predominantly Caucasian and tended to be younger than 62 years, the median age at breast cancer diagnosis in the United States (11). Our results indicate that pseudocirrhosis occurs primarily in patients who have liver metastases and are exposed to multiple lines of chemotherapy, is not associated with any particular biological subtype of breast cancer, and can be complicated by portal hypertension and hepatocellular failure. Pseudocirrhosis adversely impacted the ability of patients to tolerate further systemic therapies for breast cancer. Hepatocellular dysfunction and cancer progression were independently predictive of increased mortality in these patients.

Pseudocirrhosis has been described most frequently in patients with liver metastases from breast cancer (12–19). The underlying pathophysiology is largely unknown, but the proposed hypotheses include: (1) regenerative hyperplasia secondary to ischemic or free-radical induced hepatocyte injury in response to systemic therapies, (2) exaggerated desmoplastic response to liver metastases, (3) scarring and capsular retraction resulting from response of liver metastases to therapy, and (4) sinusoidal occlusion caused by chemotherapy-related injury (17, 18, 20). The precise mechanisms behind its preponderance in breast cancer also remains unclear. Nearly all (97%) patients in our study had previously received systemic chemotherapy, and half (50%) had been exposed to more than two lines, suggesting that chemotherapy-induced liver injury may be critical to the pathogenesis of pseudocirrhosis. Also, 94% of patients had liver metastases and 88% had extensive involvement (>10 liver lesions) indicating that local reaction to liver metastases and their response to therapies may also contribute to the process. Interestingly, responses in liver metastases were heterogeneous at the time when pseudocirrhosis was first reported, with less than a third of patients experiencing shrinkage of lesions, suggesting that tumor contraction and the resultant desmoplastic fibrosis are not essential for its causation (13). The distribution of hormone receptor and HER2 phenotypes in this study were comparable to breast cancer in the general population (11). Most patients had been treated with endocrine therapies, and the median duration of exposure was 35 months. Notably, steatohepatitis has been reported in up to 2% of patients treated with tamoxifen, particularly in the setting of co-existent obesity – a phenomenon attributed to increased hepatic lipid accumulation and oxidative stress (21, 22). Pseudocirrhosis with liver failure has also been described in two patients treated with palbociclib (23). Two groups have separately reported pseudocirrhosis in patients with hormone-receptor-positive metastatic breast cancer treated with only hormonal agents (18, 24). The current study was not designed to analyze the differential impact of various systemic therapies on the pathogenesis of pseudocirrhosis.

Despite being fundamentally different from true cirrhosis, pseudocirrhosis can lead to some shared complications, consequent to structural and functional alterations in the liver. We identified portal hypertension in 36% of patients, the most frequent manifestation being ascites with high SAAG (35%). Sixteen patients had varices on upper endoscopy and eight experienced bleeding from their rupture requiring endoscopic intervention. However, the true prevalence of varices could not be assessed since most patients did not undergo upper endoscopy. Engelman et al., in a series of 48 patients with pseudocirrhosis, identified portal hypertension in 40%, while Oliai et al. reported a prevalence of 11% among 37 patients, although they employed different diagnostic criteria (25, 26). For the purposes of this study, we defined portal hypertension as the detection of one or more of its sequelae, i.e., new splenomegaly, high-SAAG ascites, and/or varices – a definition more aligned with the former study (26). Using similar criteria, Qayyum et al. identified portal hypertension in 6 of 16 patients with diffuse hepatic nodularity following chemotherapy for metastatic breast cancer (3). The above studies reported higher rates of ascites (68-81%) than us, though they did not employ SAAG to accurately classify the ascites (25, 26). As elaborated in **Table 3**, hepatic synthetic function was not adversely impacted at the diagnosis of pseudocirrhosis in a majority of our patients. However, nearly half developed signs of hepatocellular failure during follow-up, the most frequent being direct hyperbilirubinemia (48%) and new onset of coagulopathy (30%). However, hyperbilirubinemia, hypoalbuminemia, and coagulopathy may be confounded by other etiologies such as cancer-associated cachexia, malnutrition, liver infiltration by metastases, and injury from systemic therapies (26, 27).

**Table 3:** Laboratory analyses at the time of diagnosis of pseudocirrhosis (n = 86).

Test	Median (IQR)
Total bilirubin (mg/dL)	0.5 (0.3-0.9)
Serum albumin (g/dL)	3.6 (3.2-4.0)
Aspartate transaminase (IU/L)	53.0 (34.2-114.0)
Alanine transaminase (IU/L)	33.5 (22.0 -64.7)
Alkaline phosphatase (IU/L)	165.0 (106.0-327.0)
International Normalized Ratio	1.1 (1.0-1.3)
Hemoglobin (g/dL)	11.3 (10.0-12.9)
Platelets (x 10³/mm³)	195 (137-217)

Forty patients in this study required a change in chemotherapy and 16 required a change in endocrine therapy within 4 weeks of diagnosis of pseudocirrhosis. More remarkably, 21 patients had to permanently discontinue chemotherapy, 13 had to discontinue endocrine therapy, and 17 had to be transitioned to best supportive care only. Among the 21 patients who had to permanently discontinue chemotherapy, 16 (76%) were attributable to hepatocellular dysfunction from pseudocirrhosis, 3 (14%) to declining performance status due to concurrent cancer progression, and 2 (10%) to both. These findings indicate a marked impact on the affected patients’ ability to tolerate further systemic therapies for breast cancer.

Median OS from the diagnosis of distant metastases was 51 months, with a 5-year OS of 40%, in the current study. Though numerically higher than the 5-year OS rate for metastatic breast cancer (28%) in the Surveillance, Epidemiology, and End Results (SEER) database, our study was limited by the lack of a matched control group for comparison (28). Oliai et al. had previously reported inferior OS from the time of metastatic disease for patients with pseudocirrhosis compared to those who did not develop this complication (25). In our study, survival was shorter among patients who developed hepatocellular failure, though portal hypertension did not appear to exert a similar impact. Multivariate analysis also confirmed that coagulopathy, hyperbilirubinemia, and hypoalbuminemia, presumably secondary to progressive hepatocellular dysfunction, significantly compromised survival. Intriguingly, age, race, receptor phenotype, characteristics of liver metastases, prior treatments, and manifestations of portal hypertension did not significantly impact survival.

Our study had several limitations. This was a retrospective observational study and cannot be used to determine causality. The lack of a control group limits our ability to derive conclusions about factors predisposing to pseudocirrhosis and to compare survival outcomes. Though images of the study patients were reviewed by our team, independent verification of abdominal imaging performed in all patients with metastatic breast cancer could have led to increased and earlier detection of pseudocirrhosis since these findings are often under-reported. Finally, histological examination was not performed to identify pathological correlates to imaging findings.

In conclusion, pseudocirrhosis occurs predominantly in patients with liver metastases and is often complicated by portal hypertension and hepatocellular failure. It can adversely impact the ability of patients to tolerate systemic therapies for breast cancer. Development of hepatocellular failure portends an increased risk of mortality.

## Data Availability Statement

The raw data supporting the conclusions of this article will be made available by the authors, without undue reservation.

## Ethics Statement

The studies involving human participants were reviewed and approved by Cleveland Clinic Institutional Review Board. Written informed consent for participation was not required for this study in accordance with the national legislation and the institutional requirements.

## Author Contributions

DG compiled the data as well as images and performed the statistical analyses. DG and AS wrote the manuscript. AP reviewed the images. JA proposed the concept, data acquisition, and interpretation. AP and JA made substantial edits to the manuscript. All authors contributed to the article and approved the submitted version.

## Conflict of Interest

The authors declare that the research was conducted in the absence of any commercial or financial relationships that could be construed as a potential conflict of interest.
